# Crossed Aphasia and Visuo-Spatial Neglect Following a Right Thalamic Stroke: A Case Study and Review of the Literature

**DOI:** 10.1155/2008/905187

**Published:** 2008-12-18

**Authors:** Lieve De Witte, Jo Verhoeven, Sebastiaan Engelborghs, Peter P. De Deyn, Peter Mariën

**Affiliations:** ^1^Department of LinguisticsVrije Universtiteit BrusselBrusselBelgium; ^2^Department of Communication SciencesCity UniversityLondonUK; ^3^Institute of Behavioural NeuroscienceAntwerpBelgium; ^4^Department of Neurology and Memory ClinicMiddelheim General Hospital (ZNA)AntwerpBelgium; ^5^Laboratory of Neurochemistry and BehaviorInstitute Born-Bunge FoundationUniversity of AntwerpAntwerpBelgium; ^6^Department of Health Care SciencesUniversity College AntwerpAntwerpBelgium; ^7^Department of Nursing SciencesFaculty of MedicineUniversity of AntwerpAntwerpBelgium

**Keywords:** Crossed aphasia, subcortical to aphasia, thalamus, right hemisphere, visuospatial neglect, stroke

## Abstract

Crossed aphasia in dextrals (CAD) following pure subcortical lesions is rare. This study describes a right-handed patient with an ischemic lesion in the right thalamus. In the post-acute phase of the stroke, a unique combination of ‘crossed thalamic aphasia’ was found with left visuo-spatial neglect and constructional apraxia. On the basis of the criteria used in Mariën et al. [67], this case-report is the first reliable representative of vascular CAD following an isolated lesion in the right thalamus. Furthermore, this paper presents a detailed analysis of linguistic and cognitive impairments of ‘possible’ and 'reliable' subcortical CAD-cases published since 1975. Out of 25 patients with a pure subcortical lesion, nine cases were considered as ‘possibly reliable or reliable’. A review of these cases reveals that: (1) demographic data are consistent with the general findings for the entire group of vascular CAD, (2) the neurolinguistic findings do not support the data in the general CAD-population with regard to (a) the high prevalence of transcortical aphasia and (b) the tendency towards a copresence of an oral versus written language dissociation and a ‘mirror-image’ lesion-aphasia profile, (3) subcortical CAD is not a transient phenomenon, (4) the lesion-aphasia correlations are not congruent with the high incidence of anomalous cases in the general CAD-population, (5) neuropsychological impairments may accompany subcortical CAD.

## 1. Introduction

At the end of the 19th century, the so-called Broca's doctrine assigned left hemisphere dominance for language to dextrals and right hemisphere dominance to sinistrals. As an exception to this dogma, Byrom Bramwell [5] introduced the term crossed aphasia (CA) to denote any aphasic syndrome resulting from a cerebral lesion 'ipsilateral' to the dominant hand. Bramwell considered this exceptional phenomenon as a transient condition in right-handers. However, during the following decades several studies documented persistent aphasia in dextrals due to a right hemisphere lesion [22–25,48,58]. When studies with left-handed aphasic patients in the 1950s [26,42] demonstrated that crossed

ISSN 0953-4180/08/$17.00 © 2008 – IOS Press and the authors. All rights reserved aphasia in left-handers is the rule and not the exception, crossed aphasia became the synonym for 'crossed aphasia in dextrals' (CAD).

Many hypotheses have been put forward to explain this exceptional anomalous lateralisation of language in the brain. The most frequent explanations of CAD in the earlier literature are hidden sinistrality [19,33,47, 84], absence of decussation of the pyramidal tract [48], familial left-handedness [6,21], bilateral hemispheric language representation [35,50,74] and undetected damage of the left hemisphere [22,24,38,69].

The view on crossed aphasia of the French neurologist Pierre Marie [65], who introduced the concept of subcortical aphasia at the beginning of the $20^{th}$ century, was fundamental [64–66]. He argued against 'the over-emphasised role of the cortex in speech and language dysfunctions' by stating that lesions in the striato-capsular region, particularly of the lenticular nucleus, often cause aphasic symptoms in CAD-patients irrespective of the hemisphere involved. The findings of Ardin-Delteil et al. [58], Marinesco et al. [22] and Holmes and Sadoff [29] supported this hypothesis by attributing aphasia in their patients to a lesion of the lenticular nucleus. Marie speculated that the increased incidence of pure deep lesions in the group of crossed aphasics was due to a weaker functional lateralisation of the subcortical structures. Habib, Joanette, Ali-Cherif and Poncet [41] reintroduced this idea several decades later. Because of the lack of support for this hypothesis in population studies, Laiacona, Capitani, Stangalino and Lorenzi [42] compared the incidence of deep lesions in crossed and standard aphasia. Laiacona et al. largely based their analysis of crossed aphasic cases on the review of Joanette, Puel, Nespoulos and Rasid [87] and selected the group of standard aphasia patients from the Milan aphasia database of Anna Basso (post-doctoral dissertation of Laiacona, 1985) and the German survey of Willmes and Poeck [36]. Contrary to the hypothesis of Habib et al. [41], Laiacona et al. [42] concluded that the 'prevalence of deep lesions with or without cortical involvement was not higher in crossed aphasia than in standard aphasia (47.22% versus 48.23%)'.

In their review Mariën, Paghera, De Deyn and Vignolo [66] revisited 152 vascular CAD-cases reported in the literature between 1975 and 2003. In order to identify the cases suitable for reliable research, they developed a set of diagnostic criteria for 'vascular CAD' in adults. For the purpose of this paper we added all new cases published after 2003 til 2007 applying the same set of criteria.

The aim of this article is: 1) to report the neurolinguistic and neurocognitive findings in a right-handed patient who incurred CAD following a vascular lesion of the right thalamus and 2) to critically analyse CAD in adults following a pure subcortical lesion.

## 2. Case report

L.C. is a 70-year-old right-handed nurseryman with seven years of formal education. Medical antecedents consisted of diabetes mellitus type II and paroxysmal atrial fibrillation. He was admitted to the neurological department of our hospital after sudden onset of left-sided weakness, a mild left facial nerve paresis and language disturbances. On admission, clinical neurological examination revealed a co-operative patient with a left hemiparesis, a mild left facial nerve paresis of central origin, dysarthria as well as left visuo-spatial neglect. Examination of coordination by finger-to-nose and hiel-to-knee tests disclosed dysmetria of the left arm and leg that could be explained by muscular weakness. Comprehension was severely disturbed. The patient could not execute simple verbal commands. Speech was incomprehensible as it consisted of semantic jargon. A left homonymous hemianopsia was found on Goldmann perimetry. A CT-scan of the brain on admission did not reveal any structural abnormalities. Four days after admission repeat CT showed a right thalamic lesion. One month post-onset of neurological symptoms, magnetic resonance imaging of the brain (MRI) confirmed an infarction in the median region of the right thalamus. In addition some small hypointense lesions were found surrounding the anterior and posterior lateral ventricles (Fig. 1). A $99^{m}$Tc –ethyl cysteinate (ECD) SPECT was performed 13 months poststroke using a Trionix (Ohio, USA) Triad three-detector gamma camera equipped with high resolution fan beam collimators. The projection data were reconstructed by a filtered backprojection using a Butterworth filter (with cut-off frequency of 0.7 cyc/cm and roll-off 5) resulting in trans-axial images with a pixel size of 3.56 mm. In comparison to normal database findings the quantified baseline ECD SPECT study showed a significantly decreased perfusion in the right fronto-temporo-parieto-occipital region and a severe hypoperfusion in the right thalamus (Fig. 2).

The strong right-handedness of the patient was confirmed by a laterality quotient of +100 on the Edinburgh Handedness Questionnaire [75]. After careful inquiry, no family history of left-handedness could be

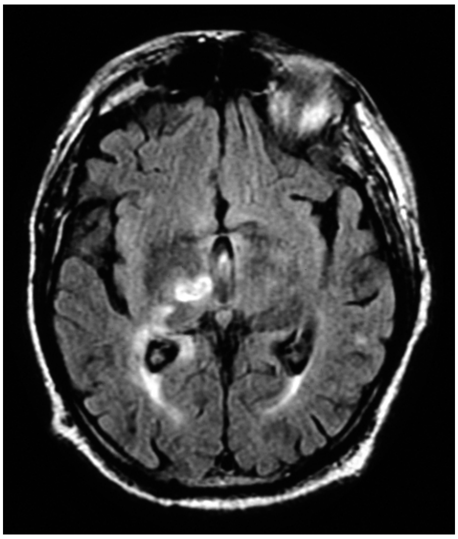

Fig. 1. Brain MRI axial FLAIR slices performed 1 month after stroke disclosing an infarction in the median region of the right thalamus as well as some small hyperintense lesions around the anterior and posterior lateral ventricles.

established. In addition, the patient had no history of brain injury or epileptic seizures and developmental milestones were entirely normal.

### 2.1. Neurocognitive examination

An in-depth neurolinguistic and neuropsychological assessment was carried out in the lesion phase (3 months post-onset) and the late phase (12 months and 18 months post-onset) of the stroke [18].

Formal language investigations consisted of standardised tests among which: the Aachen Aphasia Test (AAT) [62], the Token Test (TT) [15], the Boston Naming Test (BNT) [16,68] and a semantic verbal fluency task in which the patient had to name as many animals, clothes, vegetables and means of transport as possible within one minute (unpublished norms).

Cognitive functions were formally assessed by means of the Mini Mental State Examination (MMSE) [57], the Hierarchic Dementia Scale (HDS) [49], the Coloured Progressive Matrices (CPM) [28], the revised Wechsler Memory Scale (WMS-R) [12], the Rey Osterrieth Complex Figure Test (CFR) [71] and the Birmingham Object Recognition Battery (BORB) [51].

#### 2.1.1. Neurolinguistic investigations

As shown in Table 1, AAT results for total *language comprehension* were severely defective in the lesion phase in terms of both auditory and written comprehension. Subtest results additionally revealed a discrepancy between word and sentence comprehension. On these subtests, comprehension was strongly related to the degree of semantic and morphosyntactic complexity: results were worse on more complex instructions.

During the subsequent months, language comprehension gradually improved but still remained defective when tested 12 months poststroke.

One month post-onset *oral language* investigations showed fluent speech characterized by paragrammatic semantic jargon in which prolonged pauses indicated word-finding difficulties. On the AAT confrontation naming tests, a defective global score of 62/120 was found. The patient obtained a normal result for the naming of colours. Naming of simple nouns and compound nouns were severely deficient. Most errors were of the semantic type (8/13 errors): semantic paraphasias (e.g. vlam [flame] for kaars [candle]), semantic neologisms (e.g. ‘oliemuts’ [oil cap] for muts [cap] and onomatopoeias (e.g. ‘tikketikketik’ for ‘schrijfmachine’ [typewriter]).

Twelve months post-onset, a remission of paragrammatic semantic jargon was observed in spontaneous speech. In addition, a normal result was found on the AAT naming subtest ‘simple nouns’. Naming of compound nouns improved but had not normalised. Defective test scores on the BNT at 12 months and 18 months poststroke confirmed the persistence of word-finding difficulties the late phase. At 12 months post-onset, the BNT error profile was dominated by semantic paraphasias (8/35 errors) (e.g. stoel [chair] = bank [bench]; kantoor [office] = huis [house]; muis [mouse] = bever [beaver]), semantic neologisms (5/35) (e.g. ‘meetboog’ [measure bow] = gradenboog [protractor]; ‘ijshuis’ [ice cottage] = iglo[o]) and circumlocutions (8/35) (e.g. het woont in het water en heeft veel poten [it lives in the water and has many legs] = inktvis [octopus]). In a semantic word fluency task the patient only produced 21 correct items. On the AAT-sentence construction subtest, he obtained a deficient score of 10/30. As demonstrated by the AAT results, repetition was normal.

Test performance at 18 months poststroke showed a decline of semantic errors (21/35 = 60%; 9/26 = 34,61%). However re-examination of the BNT and the semantic word fluency task still revealed pathological scores. A normal score on the sentence construction subtest was found.

Scores on the AAT-subtests of *written language* were severely disrupted in the lesion phase. Deficient scores

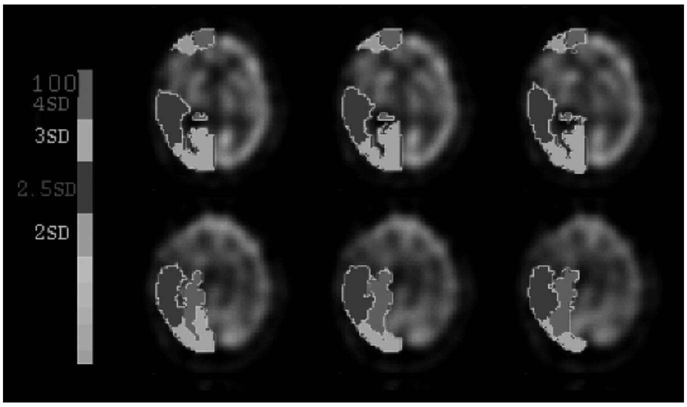

Fig. 2. Quantified Tc-99m-ethyl cysteinate dimmer SPECT performed 13 months post-onset revealing a relative cortical hypoperfusion in the fronto-temporo-parietal region associated with a marked aperfusion of the right thalamus.

were found for 'reading aloud' and 'composing on dictation'. Writing to dictation was characterized by neologistic jargonagraphia. During the late phase, writing had significantly improved: the patient obtained scores within the normal range for the AAT subtests 'composing' and 'writing to dictation'. Reading aloud remained impaired.

From the neurolinguistic assessment presented above, it can be concluded that the aphasia profile was consistent with a taxonomic diagnosis of transcortical sensory aphasia in the lesion phase of the stroke: speech was fluent with a high incidence of semantic and paragrammatic errors. In addition, language comprehension deficits, word-finding difficulties, lexical and graphical disturbances were found but repetition was intact. In the late phase, language comprehension, word-finding and reading had improved but remained defective. Only for writing, normal scores were obtained.

### *2.1.2. Neuropsychological investigations*

As shown in Table 2 a severely deficient score of 18/30 was found on the MMSE [76]. The HDS revealed concentration deficits, a short-term memory disturbance, acalculia, left visual neglect, visuo-gnostic problems and ideomotor, ideational and constructional apraxia. The WMS-R revealed a pathological verbal memory index. Because of the left visuo-spatial neglect, assessment of visual memory functions was not carried out. The patient failed to copy the complex Figure of Rey. Severe left visuo-spatial neglect was also demonstrated on the BORB subtests 'length match task', 'size match task' and 'position of gap match task'.

Cognitive functions were formally re-investigated at 12 and 18 months post-onset. The MMSE score had improved but still remained defective. Verbal memory had normalized. The patient obtained percentile 40 on the CPM. The results on the HDS-subtests assessing 'concentration', 'registration', 'remote memory', 'arithmetics', 'gnosis' and 'ideational praxis' had also normalized. However ideomotor and constructional apraxia persisted. Copying the Rey-Osterrieth Figure was deficient. Re-evaluation of the visuo-spatial functions via the BORB, however, demonstrated remission of left visuo-spatial neglect.

The above-mentioned neurocognitive investigations initially revealed severe left visuo-spatial neglect in association with visuo-gnostic disturbances, constructional apraxia, ideomotor apraxia, ideational apraxia, acalculia, disturbance of concentration and verbal memory. With the exception of ideomotor and constructional apraxia, the cognitive deficits resolved after 12 months.

## *2.2. Discussion*

Following a right thalamic ischemic infarction this patient presented in the lesion phase of the stroke a linguistic syndrome consistent with the diagnosis of crossed transcortical sensory aphasia with neologistic jargon agraphia and alexia. In association with these aphasic disturbances, severe left visuo-spatial neglect was found in conjunction with constructional, ideomotor and ideational apraxia. Although spontaneous speech had normalised at 18 months post-stroke, formal neuropsychological testing still revealed word-finding difficulties, alexia, ideomotor and constructional apraxia. With the exception of alexia, the neu-

Table 1
Neurolinguistic test results in the lesion and late phase of the stroke

| Neurolinguistic tests | month 1 | month 12 | month 18 | percentiles 1/12/18 | max | mean | SD | ASD month 1 | ASD month 12 | ASD month 18 |
|---|---|---|---|---|---|---|---|---|---|---|
| *AKENSE APHASIA TEST* | | | | | | | | | | |
| Language comprehension total | 41 | 67 | – | 4/24/- | 120 | 108.5 | 10.24 | −6.59 | −4 | – |
| auditory word comprehension | 21 | – | – | 49/–/– | 30 | 26.49 | 3.30 | −1.66 | – | – |
| auditory sentence comprehension | 14 | – | – | 16/–/– | 30 | 26.79 | 3.41 | −3.75 | – | – |
| total auditory comprehension | 35 | 35 | – | 26/26/– | 60 | 53.28 | 6.08 | −3 | −3 | – |
| reading word comprehension | 6 | – | – | 3/–/– | 30 | 28.30 | 2.29 | −9.74 | – | – |
| reading sentence comprehension | 0 | – | – | 1/–/– | 30 | 26.91 | 3.39 | −7.94 | – | – |
| total reading comprehension | 6 | 32 | – | 2/26/– | 60 | 55.21 | 4.90 | −10.04 | −4.74 | – |
| Token Test – number of errors | 34 | 4 | – | 49/94/– | 0 | | | | | – |
| Spontaneous speech | | | | | | | | | | |
| communicative behavior | – | 5 | – | | 5 | 4.63 | 0.54 | | | – |
| articulation and prosody | – | 5 | – | | 5 | 4.63 | 0.67 | | | – |
| automatisms | – | 5 | – | | 5 | 4.59 | 0.65 | | | – |
| semantic structure | – | 5 | – | | 5 | 4.59 | 0.53 | | | – |
| phonematic structure | – | 5 | – | | 5 | 4.54 | 0.56 | | | – |
| syntactic structure | – | 5 | – | | 5 | 4.41 | 0.55 | | | – |
| Imposed speech | | | | | | | | | | |
| Repetition total | 150 | 149 | – | 100/78/– | 150 | 144.1 | 8.07 | 0.73 | 0.61 | – |
| phonemes | 30 | 30 | – | 88/88/– | 30 | 28.91 | 2.09 | | | – |
| one-syllable words | 30 | 30 | – | 93/93/– | 30 | 29.22 | 1.32 | | | – |
| words of foreign origin | 30 | 30 | – | 95/95/– | 30 | 28.94 | 2.31 | | | – |
| compound words | 30 | 30 | – | 98/98/– | 30 | 28.45 | 2.22 | | | – |
| sentences | 30 | 29 | – | 99/96/– | 30 | 28.55 | 1.90 | | | – |
| Naming total | 62 | 110 | – | 38/96/– | 120 | 109.3 | 8.42 | −5.62 | 0.08 | – |
| simple nouns | 16 | 30 | – | 30/97/– | 30 | 27.92 | 2.90 | −4.11 | 0.72 | – |
| colors | 26 | 28 | – | 70/86/– | 30 | 27.69 | 1.99 | 0.85 | 0.15 | – |
| compound nouns | 10 | 23 | – | 34/69/– | 30 | 28.04 | 2.61 | −6.91 | −1.93 | – |
| sentence construction | 10 | 29 | – | 38/99/– | 30 | 25.69 | 3.72 | −4.21 | 0.89 | – |
| Written language total | 5 | 78 | – | 11/80/– | 90 | 85.52 | 7.63 | −10.55 | 0.98 | – |
| reading aloud | 4 | 24 | – | 15/56/– | 30 | 28.95 | 1.93 | −12.93 | −2.56 | – |
| composing on dictation | 1 | 28 | – | 20/89/– | 30 | 28.57 | 2.75 | −10.03 | 0.21 | – |
| writing on dictation | 0 | 26 | – | 10/83/– | 30 | 28 | 3.67 | | 0.54 | – |
| *BOSTON NAMING TEST* | – | 25 | 34 | | 60 | 51.60 | 5.87 | – | −4.53 | −2.99 |
| *VERBAL FLUENCY- semantic* | – | 21 | 29 | | | 43.40 | 11.76 | – | −1.9 | −1.20 |
| animals | | 6 | 8 | | | | | | | |
| vegetables | | 5 | 8 | | | | | | | |
| clothing | | 3 | 6 | | | | | | | |
| means of transport | | 7 | 7 | | | | | | | |

rolinguistic characteristics are semiologically compatible with the typical description of ‘non-crossed thalamic aphasia’ which is characterized by a fluent jargon aphasia in combination with hypophonia, normal repetition, normal lexical and graphical skills, auditory comprehension problems and word-finding difficulties (perseverations, neologisms, semantic and phonological paraphasias) [3,13,14,30,39,53,55,80,82]. Consequently, the anatomo-clinical configurations are compatible with ‘mirror-image’ CAD reflecting the expected lesion-behavior correlations [52,86].

The presence of ideomotor, ideational and constructional apraxia also corresponds with the neuropsychological expectations following a thalamic lesion of the dominant hemisphere [73]. Left visuo-spatial neglect in this patient corroborates the general assumption that extrapersonal and personal neglect follows from a non-dominant thalamic lesion. As a result it seems that the right thalamus in this patient subserves both typical dominant and non-dominant cognitive functions.

## 3. Further analysis of subcortical CAD

The structural lesion in our patient with CAD was located at the subcortical level in the right thalamus and was accompanied by a significantly decreased right hemisphere perfusion. Given the disruption of blood perfusion at the cortical level as evidenced by SPECT, the cognitive and linguistic dysfunctions cannot not easily be attributed exclusively to malfunctioning of subcortical structures. CAD was investigated in detail

Table 2
Neurocognitive test results in the lesion and late phase of the stroke

| Neurocognitive tests | month 1 | month 12 | month 18 | max | mean | SD | ASD month 1 | ASD month 12 | ASD month 18 |
|---|---|---|---|---|---|---|---|---|---|
| *Mini mental state examination* | 18 | 24 | – | 30 | 29 | 1.3 | − 8.46 | − 3.85 | – |
| *Hierarchic dementia scale (HDS)* | | | | | | | | | |
| *Total score* | 140 | 173 | 190 | 200 | | | | | |
| item 1: orienting | 10 | 10 | 10 | 10 | 10 | 0 | | | |
| item 2: prefrontal | 10 | 10 | 10 | 10 | 10 | 0 | | | |
| item 3: ideomotor | 8 | 8 | 8 | 10 | 9.89 | 0.31 | − 6.10 | − 6.10 | − 6.10 |
| item 4: looking | 6 | 8 | 10 | 10 | 10 | 0 | − 4 | − 2 | − 2 |
| item 5: ideational | 8 | 10 | 10 | 10 | 9.98 | 0.15 | − 13.2 | 0.13 | 0.13 |
| item 6: denomination | 4 | 5 | 10 | 10 | 9.89 | 0.31 | − 31.9 | − 15.78 | 0.35 |
| item 7: comprehension | 5 | 9 | 9 | 10 | 9.93 | 0.25 | − 19.72 | − 3.72 | − 3.72 |
| item 8: registration | 6 | 8 | 10 | 10 | 9.67 | 1.01 | − 3.63 | − 1.65 | 0.33 |
| item 9: gnosis | 7 | 8 | 9 | 10 | 9.80 | 0.40 | − 7 | − 4.5 | − 2 |
| item 10: reading | 2 | 5 | 6 | 10 | 9.78 | 0.63 | − 12.34 | − 7.59 | − 6 |
| item 11: orientation | 10 | 10 | 10 | 10 | 9.93 | 0.25 | | | |
| item 12: construction | 4 | 10 | 10 | 10 | 9.35 | 1.27 | − 4.21 | 0.51 | 0.51 |
| item 13: concentration | 5 | 9 | 9 | 10 | 9.11 | 0.90 | − 4.57 | − 0.12 | − 0.12 |
| item 14: calculation | 5 | 9 | 9 | 10 | 9.11 | 0.96 | − 4.28 | − 0.11 | − 0.11 |
| item 15: drawing | 6 | 6 | 10 | 10 | 9.24 | 0.90 | − 3.60 | − 3.60 | 0.84 |
| item 16: motor | 10 | 10 | 10 | 10 | 9.61 | 1.34 | | | |
| item 17: remote memory | 10 | 10 | 10 | 10 | 9.91 | 0.59 | | | |
| item 18: writing | 6 | 10 | 10 | 10 | 9.72 | 0.58 | − 6.41 | 0.48 | 0.48 |
| item 19: similarities | 8 | 10 | 10 | 10 | 9.26 | 1.22 | − 1.03 | 0.61 | 0.61 |
| item 20: recent memory | 10 | 8 | 10 | 10 | 9.65 | 0.77 | | | |
| *Wechsler memory scale (WMS-R)* | | | | | | | | | |
| *Visual Memory Index* | | impossible | | | | 100 | 15 | | |
| *Verbal Memory Index* | 68 | 84 | – | | 100 | 15 | − 2.13 | − 1.01 | – |
| *Global Memory Index* | – | – | – | | 100 | 15 | | | |
| logical memory (A + B) | 22 | 26 | – | | 26 | | | | |
| verbal paired associates (I–III) | 3 | 7 | – | | | | | | |
| *Rey Osterrieth figure (Copy)* | – | 12 | 19 | | 36 | 25 | 3 | | |
| *BORB* | | | | | | | | | |
| length match task – A | 12 | 29 | – | 30 | 26.9 | 1.6 | − 9.3 | 1.31 | – |
| size match task – A | 17 | 28 | – | 30 | 27.3 | 2.4 | − 4.29 | 0.29 | – |
| orientation match task – A | 23 | 24 | – | 30 | 24.8 | 2.6 | − 0.69 | − 0.31 | – |
| position of gap match task – A | 22 | 36 | – | 40 | 35.1 | 4 | − 3.28 | 0.22 | – |
| minimal feature match | 13 | 24 | – | 25 | 23.3 | 2 | − 5.15 | 0.35 | – |
| foreshortened match | – | 24 | – | 25 | 21.6 | 2.6 | – | 0.92 | – |
| object decision | – | 26 | – | 32 | 27 | 2.2 | – | − 0.45 | – |
| *Raven colored progressive matrices* | – | – | 19 | 36 | pc 40 | | | | |

in Mariën et al. [67], who systematically studied 152 CAD cases reported in the literature between 1975 and 2003. However, Mariën et al. [67] did not separately investigate the neurolinguistic and neuropsychological impact of subcortical lesions in CAD. In the remainder of this paper, the Mariën 2004-corpus is extended by all CAD cases reported between 2003 and 2007 (abovementioned patient inclusive) and the neurolinguistic and nonverbal cognitive impairments associated with subcortical lesions will be reviewed.

### *3.1. Methods*

Given the rarety of CAD, we are inclined to follow the plea of Coppens and Hungerford [61] to avoid unnecessarily strict inclusion criteria. However, less stringent diagnostic criteria render several case-reports ambiguous and hence useless for drawing general conclusions, in spite of the often illuminating insights presented in the discussion of these cases. Therefore, it seems appropriate to adopt the more rigorous algorithm, defining the diagnostic criteria for CAD, as described by Mariën et al. [67] for the classification of 'vascular CAD' in adults. This algorithm is based on the following 5 criteria: 1) clear-cut evidence of aphasia, 2) evidence of natural (i.e. not shifted) right-handedness, documented by a formal test, 3) evidence of lesions strictly confined to the right hemisphere, leaving the left hemisphere structurally intact, 4) absence of familial left-handedness or ambidexterity, and 5) no his-

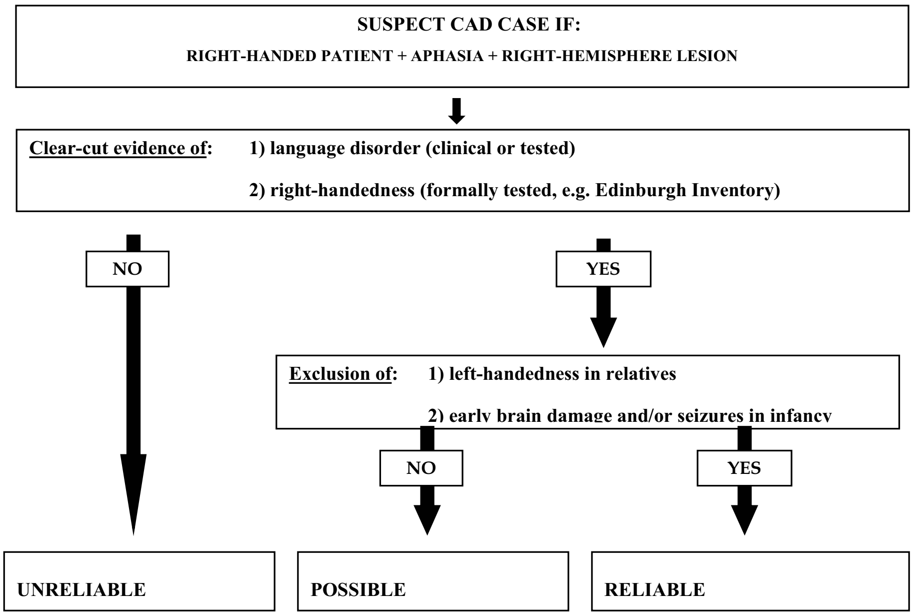

Fig. 3. Algorithm of diagnostic criteria for vascular CAD in adults [67].

tory of early brain damage and/or seizures in childhood (Fig. 3). On the basis of this algorithm, Mariën et al. [67] classified 152 vascular CAD-cases for the period 1975–2003 into three categories: 1) unreliable CAD-cases ($n$ = 85), 2) possible CAD-cases ($n$ = 18) and 3) reliable CAD-cases ($n$ = 49). For the present investigation, 23 vascular cases were added who were reported between 2003 and 2007 (this patient inclusive) and the same criteria were applied as in Mariën et al. [67] (cfr. Appendix 1: classification of adults CAD-cases with a vascular etiology 1975–2007).

From this corpus of 176 cases, only the CAD-cases with pure subcortical lesions were selected for further analysis. Subcortical lesion localisation was based on structural brain imaging data (CT/MRI). Unreliable subcortical CAD-cases were excluded from further investigation because of: 1) absence of clear-cut evidence of right-handedness ($n$ = 15) (case nrs. 1–3, 5–10, 12, 16–18, 20, 21) or 2) uncertainty about the structural integrity of the left hemisphere ($n$ = 1) (case nr. 24) (see Appendix 2). A detailed analysis of neurolinguistic and nonverbal cognitive impairments was carried out in the group of 'possible' ($n$ = 2) (case nrs. 11, 15) and 'reliable' ($n$ = 7) (case nrs. 4, 13, 14, 19, 22, 23, 25) CAD with an isolated subcortical lesion (see Appendix 3, Appendix 4). Language was analyzed in terms of six linguistic parameters: 1) oral verbal fluency, 2) auditory-verbal comprehension, 3) repetition, 4) naming, 5) reading and 6) writing. The degree of impairment was expressed on a four-point scale (severe = + + +, moderate = ++, mild = + and none = 0). The taxonomic diagnosis of the case-reports was also checked. Whenever the case-reports lacked a taxonomic label, it was attempted to classify the type of aphasia using the six linguistic parameters mentioned above. In the quantitative analysis of aphasia, the following measures were considered: 1) total severity (range from zero to 18: 1 to 6 indicating mild aphasia, 7 to 12 moderate and 13 to 18 severe aphasia), 2) oral versus written language dissociation and 3) language recovery. Anatomo-clinical correlations were analysed in cases documented by linguistic data in the lesion phase. An attempt was made to divide the subcortical CAD-cases into 'mirror-image' and 'anomalous' cases [52,86]. Cases with a lesion-aphasia profile comparable to those following an analogous lesion in the left hemisphere were identified as 'mirror-image' cases. Cases with an unexpected lesion-aphasia correlation were considered as 'anomalous' cases. An analysis of nonverbal disorders in a time frame model was not possible because the original case-reports did not systematically describe the deficits on a temporal basis. Consequently, analysis was restricted to a list marking the absence or presence of a deficit.

### 3.2. Results

On the basis of the paradigm developed in Mariën et al., 97 out of 176 cases (= 55.1%) were identified as ‘unreliable CAD’, 22 (= 12.5%) were considered as ‘possible CAD’ and 57 (= 32.4%) emerged as ‘reliable CAD’ (Appendix 1). Out of the 176 cases, 151 cases (= 85.8%) (case nrs. 26–176) had cortico-subcortical lesions while 25 patients (= 14.2%) (case numbers 1–25) had isolated subcortical damage (Appendix 2). Focal thalamic damage was only found in four patients (= 2.3%) (case nrs. 6, 8, 21, 25). Sixteen pure subcortical CAD cases (= 64%) were classified as ‘unreliable (case nrs. 1–3, 5–10, 12, 16–18, 20, 21, 24). Nine patients (= 36%) were identified as ‘possible’ ($n = 2$) (case nrs. 11, 15) or ‘reliable’ subcortical CAD-cases ($n = 7$) (case nrs. 4, 13, 14, 19, 22, 23, 25). The reliable subcortical CAD patients will be further analysed in terms of their demographic, neurolinguistic, and neuropsychologic characteristics.

*3.2.1. Demographic characteristics*

The nine reliable CAD cases with subcortical damage who were selected for further analysis (see Appendices 3 and 4) had a mean age of 65.5 years (range 38–79 years, SD = 12.06). Five out of 9 patients were men (= 55.5%) and four women (= 44.5%).

*3.2.2. Neurolinguistic characteristics*

Neurolinguistically, fluent aphasia ($n = 4$) (case nrs. 4, 14, 15, 25) was found almost as frequently as nonfluent aphasia ($n = 3$) (case nrs. 13, 19, 22). In two cases (case nrs. 11, 23), nonfluent aphasia was reported in the acute phase but no follow-up data were provided.

With respect to the clinical type of aphasia in the lesion phase, four patients (case nrs. 13, 19, 22, 25) presented with transcortical aphasia and one patient with Wernicke aphasia (case nr. 4). In two patients (case nrs. 14, 15) there was no indication of aphasia. The case of Deleval and Léonard (case nr. 11) presented with Broca-aphasia in the acute phase but no follow-up data were provided.

Quantitative analysis could be carried out for total severity of linguistic impairment and analysis of oral versus written language was possible in six cases (case nrs. 4, 14, 15, 19, 22 25). Out of six cases, three cases (case nrs. 11, 13, 23) were excluded because of the absence of lesion phase data.

Aphasia in the lesion phase was moderate in three cases (case nrs. 4, 22, 25) and severe in one case (case nr. 19). Two patients (case nrs. 14, 15) had no aphasia. In this small group of reliable subcortical CAD-cases, 3 patients (3/6) (case nrs. 19, 22, 25) were more severly impaired in written than oral language.

‘Language recovery’ could not be analysed because of the limited number of case-reports ($n = 4$) (case nrs. 4, 13, 22, 25) providing a complete description of the aphasia in the lesion and the late phase (Appendix 3).

As to the lesion site-aphasia correlation, four patients were excluded because of a lack of data (case nrs. 11, 23) and because of the absence of aphasia during the acute phase (case nrs. 14, 15). Out of the five remaining cases, four patients (case nrs. 13, 19, 22, 25) presented a ‘mirror-image’ type, consistent with the lesion-aphasia relationships in uncrossed aphasia. The case of Colombo et al. (case nr. 4) belongs to the ‘anomalous’ CAD group as it violates classical lesion-aphasia expectations.

*3.2.3. Neuropsychological characteristics*

As shown in Appendix 4, a variety of nonverbal disorders such as dysprosody, visual neglect, constructional, ideomotor, ideational and oral apraxia, acalculia as well as impairment of memory, orientation and nonverbal intelligence have been reported in the group of possible and reliable subcortical CAD-cases (case nrs. 4, 11, 13–15, 19, 22, 23, 25).

*3.2.3.1. Prosody*

In five (case nrs. 13, 14, 19, 22, 25) out of the nine subcortical CAD-cases, prosodic impairment was reported. Four cases (case nrs. 13, 14, 19, 22) presented dysprosodia.

*3.2.3.2. Visuo-spatial neglect*

Left-sided visuo-spatial neglect was investigated in eight patients (case nrs. 4, 11, 13-15, 19, 22, 25) of whom five (case nrs. 4, 13, 19, 22, 25) presented this disorder.

*3.2.3.3. Constructional apraxia*

Constructional apraxia was examined in six cases (case nrs. 4, 11, 15, 19, 22, 25) all of which presented this disorder with the exception of the case of Gomez-Tortosa et al. (case nr. 15).

*3.2.3.4. Ideomotor, ideational and oral apraxia*

Ideomotor praxis was assessed in eight subcortical CAD-cases (case nrs. 4, 11, 13, 14, 15, 19, 22, 25) of which three (case nrs. 4, 13, 25) demonstrated ideomotor apraxia. Ideational praxis was described in seven cases (case nrs. 4, 11, 13, 15, 19, 22, 25). In three (case nrs. 4, 19, 25) out of the seven cases, ideational apraxia was found. In seven (case nrs. 4, 13-15, 19, 22, 25) out of all subcortical CAD-cases, a specific statement was made about oral praxis. Two (case nrs. 4, 13) of them had oral apraxia.

#### 3.2.3.5. Arithmetics

Arithmetics was only investigated in two patients (case nrs. 19, 25) of whom both presented acalculia.

#### 3.2.3.6. Other cognitive domains

In five (case nrs. 4, 11, 13, 15, 25) out of the six cases (case nrs. 4,11, 13, 15, 22, 25) in which additional neuropsychological domains were investigated anosognosia, concentration and memory deficits were described.

### 3.3. Discussion

This review shows that subcortical vascular CAD ($n = 25$) occurs in 14.2% of the total corpus of vascular CAD cases ($n = 176$) published between 1975–2007. This finding strongly contradicts the hypothesis of Habib et al. [41] which postulates that pure subcortical lesions are overrepresented in the group of crossed aphasia patients. According to the paradigm of Mariën et al. [67], nine 'possible and reliable' subcortical CAD-cases were analyzed. The demographic data of this small group (mean age: 65.4; gender: 5 men, 4 women) are consistent with the general findings in the entire group of vascular CAD which show that CAD patients are not younger than non-crossed aphasics nor that CAD is more frequent in women than men [67]. In contrast with the most frequent aphasia type in the general CAD population, no Broca-like aphasia was found in subcortical CAD-cases. Transcortical aphasia was the most frequent type of aphasia in the subcortical CAD group ($n = 4/9$). This finding is consistent with the hypothesis suggesting similarity between subcortical and transcortical aphasia on the basis of the preservation of repetitive speech [2,14].

A quantitative analysis of the aphasia profiles in this study suggests some tentative conclusions. The fact that most subcortical CAD-patients (6/8, (case nrs. 4, 13, 19, 22, 25); one patient (case nr. 22) was excluded because of the lack of data in the lesion phase) were still aphasic in the lesion phase is in agreement with the findings in the general CAD-population which indicates that CAD is not a transient disorder. However, these findings contradict the general assumption that 'non-crossed subcortical aphasias' are characterised by a more rapid recovery than cortical aphasia [11,85,87] and have less severe impact on written language [45, 46]. In this review the oral versus written language dissociation was only examined in six cases ( case nrs. 4, 14, 15, 19, 22, 25). In three (case nrs. 19, 22, 25) out of six cases, written language was more severely impaired than oral language. Four cases (case nrs. 11, 13–15) had to be excluded from the lesion-behavior analysis because of the lack of data in the lesion phase and because of the normalisation of language during the acute phase.

As to the lesion-aphasia correlations, almost all subcortical CAD-cases (4/5) had a mirror-image profile (case nrs. 13, 19, 22, 25) which contrasts with the high incidence of 39.5% (15/38) of anomalous cases in the general vascular CAD-population [67]. This finding suggests a more consistent topographical organisation of neurolinguistic functions of the phylogenetically older subcortical structures. In-depth analysis of nonverbal disorders accompanying subcortical CAD was not possible since most cases were not systematically documented on a temporal basis. However, a variety of neuropsychological deficits such as dysprosody, visual neglect, constructional, ideomotor, ideational and oral apraxia, acalculia as well as impairment of memory, orientation and nonverbal intelligence have been reported in subcortical CAD-cases. Analogous to the patient reported in this paper, four of the eight subcortical CAD-cases presented the same association of crossed aphasia with left visuo-spatial neglect and apraxia (case nrs. 4, 13, 19, 22). With the exception of the first case-report of Cappa et al. [79], all cases (3/4) displayed left visuo-spatial neglect and constructional apraxia. Consequently, the combination of visuo-spatial neglect and constructional apraxia confirms the general assumption of both neglect and constructional apraxia as frequent nonverbal disorders in vascular subcortical CAD [27]. The subcortical lesion localisation of these four cases is quite different: Colombo et al. [1] recorded these symptoms after a lesion in the lentiform nucleus, while the patient of Cappa et al. [79] had a lesion in the lentiform nucleus in addition to lesions in the periventricular white matter. Mariën et al. [70] described a patient with a subcortical lesion involving the internal and external capsule, the pallidal globe, the putamen, the claustrum and the periventricular white matter. Laiacona et al. [43] reported a patient with a thalamic lesion and involvement of the internal capsule and putamen.

Among the explanations of the pathophysiological mechanism underlying reversed cerebral dominance for language in the CAD-population, Marie [64–66] and later Habib et al. [41] stated that language functions are not lateralized at the level of the subcortical structures. As a consequence of an absence of cerebral language dominance at the subcortical level, an overrepresentation of subcortical CAD was assumed [41].

In their review of subcortical CAD, Laiacona et al. [44] rejected this point of view because of the lack of confirmation of this hypothesis in population studies. As an alternative explanation, Laiacona et al. [44] proposed the view of 'crowding of functions' in the right hemisphere which implies that 'right deep structures are crucial for language and visual attention when the latter functions are both subserved by the right hemisphere'. This view was based on the fact that the incidence of crossed aphasia with unilateral neglect was higher in the subcortical than cortical CAD-group of Laiacona's study.

Our current observation in which a combination of dominant (aphasia, apraxia) and non-dominant hemisphere disorders (left visual neglect) is present, corroborates the view of ' a crowding of functions'. Moreover, stringent analysis of the reliable subcortical CAD-group resulted in almost two thirds (5/8 = 62.5%) (case nrs. 4, 13, 19, 22, 25) of patients presenting with crossed aphasia in combination with visuo-spatial neglect. Analysis of anatomo-clinical configurations in these subcortical CAD-cases ($n = 5$) reveals that four out of five mirror-image cases displayed a combination of dominant and non-dominant hemisphere symptoms (case nrs. 13, 19, 22, 25).

## 4. General conclusion

The patient described in this paper incurred an ischemic lesion in the right thalamus. In the post-acute phase of the stroke, a unique combination of 'crossed thalamic aphasia' was found with left visuo-spatial neglect and constructional apraxia. As such, this patient represents the first reliable representative of CAD following an isolated lesion in the right thalamus. A further in-depth analysis of the linguistic and cognitive impairments of CAD-patients with a pure subcortical lesion reveals that the demographic data are consistent with the general findings of the entire group of vascular CAD cases. In addition, the neurolinguistic findings do not support the data in the general CAD-population with respect to the high prevalence of transcortical aphasia and the tendency towards a copresence of an oral versus written language dissociation and a 'mirror image' lesion-aphasia profile. Furthermore, subcortical CAD does not seem to be a transient phenomenon and the lesion-aphasia correlations are not congruent with the high incidence of anomalous cases in the general CAD population. Moreover, it was observed that subcortical CAD may be accompanied by neuropsychological impairments such as dysprosody, visual neglect, apraxia, acalculia and disturbance of orientation and memory. Finally, the frequent association of 'crossed subcortical aphasia' with left visual neglect, normally attributed to a lesion in the non-dominant hemisphere, corroborates the view of a crowding of functions. However, further investigation of subcortical CAD-cases is required to uncover the mechanism underlying the cerebral dominance of the diverse neuropsychological functions.

Appendix 1: Classification of adults CAD-cases with a vascular etiology 1975–2007 ($n = 176$)

| unreliable CAD ($n = 97$) | | possible CAD ($n = 22$) | | reliable CAD ($n = 57$) | |
|---|---|---|---|---|---|
| absence clear-cut evidence of | | presence of familial sinistrality | | | |
| right handedness ($n = 83$) | | ($n = 9$) | | | |
| 1976 | Kishida et al., case 3, | 1976 | Lozano and Clark | 1977 | April and Tse |
| | Kishida et al., case 4 | | Zangwill, case 2 | 1980 | April and Han |
| | Wechsler | 1981 | Assal et al. | 1981 | Carr et al., case 2 |
| 1977 | Sadavisam & Jaganathan | 1983 | Henderson, case 1 | | Carr et al., case 3 |
| | Yamada et al. | 1984 | Demeurisse et al. | | Denes and Caviezel |
| 1978 | Urbain et al. | 1988 | Kojima et al., case 2 | 1982 | Assal |
| 1979 | Barroche et al. | 1989 | Alexander et al., case 1 | 1983 | Habib et al. |
| | Goldstein et al., case 1 | 1992 | Marshall and Halligan | | Henderson, case 2 |
| | Goldstein et al., case 2 | 1994 | Gomez-Tortosa et al. | | Henderson, case 3 |
| | Hyodo et al. | | | 1984 | Colombo et al. |
| | Pillon et al., case 1 | absence of information about | | | Hindson et al. |
| | Pillon et al., case 2 | familial sinistrality ($n = 6$) | | | Sartori et al. |
| | Tsuruoka et al., case 2 | | | 1985 | Basso et al., case 1 |
| | Tsuruoka et al., case 3 | 1987 | Rapcsak et al. = Ochipa | | Basso et al., case 2 |
| | Zangwill, case 1 | | and Gonzalez Rothi (1989) | | Basso et al., case 3 |
| 1980 | Tanabe et al. | 1991 | Deleval and Léonard, case 3 | | Basso et al., case 4 |
| | Donoso et al., case 2 | 1992 | Coppens and Robey, case 1 | | Basso et al., case 5 |
| 1981 | Yarnell, case 1 | | Coppens and Robey, case 2 | | Basso et al., case 6 |
| | Yarnell, case 2 | 1994 | Trojano et al. | | Basso et al., case 7 |
| | Yarnell, case 3 | 1996 | Nédélec-Cicéri et al. | | Sugimoto et al. |
| | Yokoyama et al., case 2 | | | 1987 | Fournet et al. |
| 1982 | Brust et al. | absence of information of early | | | Perani et al., case 1 |
| | Endo et al., case 1 | brain damage and/or seizures in | | | Walker-Batson et al. |
| | Endo et al., case 2 | infancy ($n = 7$) | | 1988 | Kojima et al., case 1 |
| | Endo et al., case 3 | | | 1989 | Nagaraja et al. |
| | Endo et al., case 4 | 1982 | Haaland and Miranda | 1990 | Faglia and Vignolo |
| | Endo et al., case 5 | 1986 | *Youngjohn* | 1991 | Berndt et al. |
| | Endo et al., case 6 | 1994 | Davous and Boller | | Deleval and Léonard, case 1 |
| | Endo et al., case 7 | 1999 | Roebroek et al. | | Deleval and Léonard, case 2 |
| | Endo et al., case 8 | 2003 | *Marangolo et al.* | | Hadar et al. |
| | Endo et al., case 9 | | *Njemanze* | 1992 | Lanoë et al. |
| | Puel et al., case 1 | 2006 | *Mansur et al.* | | Sakurai et al. |
| | Wertz | | | 1993 | Cappa et al., case 1 |
| 1983 | Donoso, case 2 | | | | Cappa et al., case 2 |
| 1984 | Kapur & Dunkley | | | | Cohen et al. |
| 1985 | Fromm et al., case 6 | | | 1994 | Berthier and Starkstein |
| | Fromm et al., case 11 | | | | Ihori et al. |
| | Mendes and Benson, case 3 | | | | Rey et al. |
| 1986 | Gonzalo, Barrio et al. | | | 1996 | Laiacona et al. |
| | Sapir et al. | | | 1997 | Fujii et al. |
| 1987 | Berthier et al. | | | 1998 | Hashimoto et al. |
| | Castro-Caldas et al., case 1 | | | 1999 | and 2001 Raymer et al. |
| | Castro-Caldas et al., case 2 | | | 2001 | Mariën et al., case 1 |
| | Castro-Caldas et al., case 3 | | | | Mariën et al., case 3 |
| | Castro-Caldas et al., case 4 | | | | Mariën et al., case 4 |
| | Gonzalez-Rothi et al | | | | Mariën et al., case 5 |
| | Hamasaki et al. | | | | Mariën et al., case 6 |
| | Klonoff et al. | | | | Mariën et al., case 7 |
| | Murdoch | | | | Mariën et al., case 9 |
| | Reinvang | | | 2002 | Paghera et al. |
| | Rétif et al., case 2 | | | 2003 | *Diouf et al.* |
| | Schweiger et al. | | | 2004 | *Bartha et al., case 1* |
| 1987 | Washima et al. | | | 2006 | *Semenza et al., case 1* |
| 1988 | Perani et al., case 2 | | | | *Semenza et al., case 2* |
| 1989 | Delreux et al. | | | | *Semenza et al., case 5* |
| | Tanridag and Ongel | | | | *Semenza et al., case 6* |

| unreliable CAD ($n = 97$) | | possible CAD ($n = 22$) | reliable CAD ($n = 57$) | |
|---|---|---|---|---|
| 1991 | Dobrzynska, case 1 | | 2007 | *current observation* |
| | Dobrzynska, case 2 | | | |
| | Ferro et al. | | | |
| 1992 | Yamadori et al. | | | |
| 1993 | Shibati et al. | | | |
| 1996 | Alexander and Annett, case 4 | | | |
| | Alexander and Annett, case 5 | | | |
| | Alexander and Annett, case 6 | | | |
| | Alexander and Annett, case 7 | | | |
| | Alexander and Annett, case 8 | | | |
| 1996 | Bakar et al., case 1 | | | |
| | Bakar et al., case 2 | | | |
| | Bakar et al., case 3 | | | |
| | Melzi et al. | | | |
| 1997 | Stefanis et al. | | | |
| 1998 | Osmon et al. | | | |
| | Ozeren et al. | | | |
| 1999 | Nagaratnam et al. | | | |
| 2000 | *Gass et al.* | | | |
| 2001 | *Coppens and Hungerford, case 1* | | | |
| | *Coppens and Hungerford, case 2* | | | |
| | *Maeshima et al.* | | | |
| | *Shintani et al.* | | | |
| 2004 | *Seddoh et al.* | | | |
| | *Sheehy & Haines* | | | |
| 2007 | *Salis et al.* | | | |
| | *Tabeling et al.* | | | |
| presence of a left hemisphere lesion ($n = 9$) | | | | |
| 1980 | Trojanowski et al. | | | |
| 1981 | Yokoyama et al., case 1 | | | |
| 1984 | Sweet et al. | | | |
| 1989 | Alexander et al., case 2 | | | |
| 1990 | Kitayama et al. | | | |
| 1992 | Le Gall et al. | | | |
| 1994 | Caramelli et al. | | | |
| 1999 | *Visch-Brink et al.* | | | |
| 2001 | Mariën et al., case 8 | | | |
| left hemisphere integrity not ascertained ($n = 4$) | | | | |
| 1981 | Carr et al., case 1 | | | |
| | Carr et al., case 4 | | | |
| 2002 | *Paparounas et al.* | | | |
| 2006 | *Bhatnagar et al.* | | | |

Legend: References printed in cursive cases added till 2007.

Appendix 2: Classification of adult CAD-cases with pure subcortical and cortico-subcortical involvement (1975–2007) ($n$ =176)

| pure subcortical involvement ($n = 25$) | | | cortico-subcortical involvement ($n = 151$) | | | | | |
|---|---|---|---|---|---|---|---|---|
| [1] | 1977 | Sadavisam and Jaganatham | [26] | 1976 | Kishida et al., case 3 | [89] | 1986 | Gonzalo, Barrio et al. |
| [2] | 1979 | Goldstein et al., case 1 | [27] | | Kishida et al., case 4 | [90] | | Sapir et al. |
| [3] | | Pillon et al., case 1 | [28] | | Lozano and Clark | [91] | | *Youngjohn* |
| [4] | 1984 | Colombo et al. | [29] | | Wechsler | [92] | 1987 | Berthier et al. |
| [5] | 1985 | Fromm et al., case 6 | [30] | | Zangwill, case 2 | [93] | | Castro-Caldas et al., case 1 |
| [6] | | Fromm et al., case 11 | [31] | 1977 | April and Tse | [94] | | Castro-Caldas et al., case 2 |
| [7] | 1987 | Hamasaki et al. | [32] | | Yamada et al. | [95] | | Castro-Caldas et al., case 3 |
| [8] | | Murdoch | [33] | 1978 | Urbain et al. | [96] | | Castro-Caldas et al., case 4 |
| [9] | | Washimi et al. | [34] | 1979 | Barroche et al. | [97] | | Fournet et al. |
| [10] | 1988 | Perani et al., case 2 | [35] | | Goldstein et al., case 2 | [98] | | Gonzalez-Rothi et al. |
| [11] | 1991 | Deleval and Léonard,case 3 | [36] | | Hyodo et al. | [99] | | Klonoff et al. |
| [12] | | Dobrzynska, case 2 | [37] | | Pillon et al., case 2 | [100] | | Rapcsak et al.=Ochipa et al. |
| [13] | 1993 | Cappa et al., case 1 | [38] | | Tsuruoka et al., case 2 | [101] | | Reinvang |
| [14] | | Cappa et al., case 2 | [39] | | Tsuruoka et al., case 3 | [102] | | Rétif et al., case 2 |
| [15] | 1994 | Gomez-Tortosa et al. | [40] | | Zangwill, case 1 | [103] | | Schweiger et al. |
| [16] | 1996 | Alexander and Annett,case 6 | [41] | 1980 | April and Han | [104] | 1988 | Kojima et al., case 1 |
| [17] | | Bakar et al., case 2 | [42] | | Tanabe et al. (japanese) | [105] | | Kojima et al., case 2 |
| [18] | | Bakar et al., case 3 | [43] | | Trojanowski et al. | [106] | | Perani et al., case 1 |
| [19] | | Laiacona et al. | [44] | | Donoso et al., case 2 | [107] | | Walker-Batson, case 1 |
| [20] | 1998 | Ozeren er al. | [45] | 1981 | Assal et al. | [108] | | Alexander et al., case 1 |
| [21] | 2001 | *Maeshima et al.* | [46] | | Carr et al., case 1 | [109] | | Alexander et al., case 2 |
| [22] | | Mariën et al., case 3 | [47] | | Carr et al., case 2 | [110] | | Delreux et al. |
| [23] | | Mariën et al., case 4 | [48] | | Carr et al., case 3 | [111] | | Nagaraja et al. |
| [24] | 2006 | Bhatnagar et al. | [49] | | Carr et al., case 4 | [112] | | Tanridag and Ongel |
| [25] | 2007 | current observation | [50] | | Denes and Caviezel | [113] | 1990 | Kitayama et al. |
| | | | [51] | | Yarnell, case 1 | [114] | | Faglia and Vignolo |
| | | | [52] | | Yarnell, case 2 | [115] | 1991 | Berndt et al. |
| | | | [53] | | Yarnell, case 3 | [116] | | Deleval and Léonard, case1 |
| | | | [54] | | Yokoyama et al., case 1 | [117] | | Deleval and Léonard, case 2 |
| | | | [55] | | Yokoyama et al., case 2 | [118] | | Dobrzynska, case 1 |
| | | | [56] | 1982 | Assal | [119] | | Ferro et al. |
| | | | [57] | | Brust et al. | [120] | | Hadar et al. |
| | | | [58] | | Endo et al., case 1 | [121] | 1992 | Coppens and Robbey,case 1 |
| | | | [59] | | Endo et al., case 2 | [122] | | Coppens and Robbey,case 2 |
| | | | [60] | | Endo et al., case 3 | [123] | | Lanoë et al. |
| | | | [61] | | Endo et al., case 4 | [124] | | Le Gall et al. |
| | | | [62] | | Endo et al., case 5 | [125] | | Marshall and Halligan |
| | | | [63] | | Endo et al., case 6 | [126] | | Sakurai et al. |
| | | | [64] | | Endo et al., case 7 | [127] | | Yamadori et al. |
| | | | [65] | | Endo et al., case 8 | [128] | 1993 | Cohen et al. |
| | | | [66] | | Endo et al., case 9 | [129] | | Shibati et al. |
| | | | [67] | | Haaland and Miranda | [130] | 1994 | Berthier and Starkstein |
| | | | [68] | | Wertz | [131] | | Caramelli et al. |
| | | | [69] | 1983 | Donoso, case 2 | [132] | | Davous and Boller |
| | | | [70] | | Habib et al. | [133] | | Ihori et al. |
| | | | [71] | | Henderson, case 1 | [134] | | Rey et al. |
| | | | [72] | | Henderson, case 2 | [135] | | Trojano et al. |
| | | | [71] | | Henderson, case 3 | [136] | 1996 | Alexander and Annett, case 4 |
| | | | [74] | 1984 | Demeurisse et al. | [137] | | Alexander and Annett, case 5 |
| | | | [75] | | Hindson et al. | [138] | | Alexander and Annett, case 7 |
| | | | [76] | | Kapur and Dunkley | [139] | | Alexander and Annett, case 8 |
| | | | [77] | | Puel et al., case 1 | [140] | | Bakar et al., case 1 |
| | | | [78] | | Sartori et al. | [141] | | Melzi et al. |
| | | | [79] | | Sweet et al. | [142] | | Nédélec-Cicéri et al. |
| | | | [80] | 1985 | Basso et al., case 1 | [143] | 1997 | Fujii et al. |
| | | | [81] | | Basso et al., case 2 | [144] | | Stefanis et al. |
| | | | [82] | | Basso et al., case 3 | [145] | 1998 | Hashimoto et al. |

| pure subcortical involvement ($n = 25$) | cortico-subcortical involvement ($n = 151$) | | | | | |
|---|---|---|---|---|---|---|
| | [83] | | Basso et al., case 4 | [146] | | Osmon et al. |
| | [84] | | Basso et al., case 5 | [147] | 1999 | Nagaratnam et al. |
| | [85] | | Basso et al., case 6 | [148] | | Raymer et al. (2001) |
| | [86] | | Basso et al., case 7 | [149] | | Roebroek et al. |
| | [87] | | Mendes and Benson, case 3 | [150] | | *Visch-Brink et al.* |
| | [88] | | Sugimoto et al. | [151] | 2000 | *Gass et al.* |
| | [152] | 2001 | *Coppens and Hungerford, case 1* | [165] | | *Marangola et al.* |
| | [153] | | *Coppens and Hungerford, case 2* | [166] | 2003 | *Njemanze et al.* |
| | [154] | | Mariën et al., case 1 | [167] | | *Semenza et al., case 1* |
| | [155] | | Mariën et al., case 2 | [168] | | *Semenza et al., case 2* |
| | [156] | | Mariën et al., case 5 | [169] | | *Semenza et al., case 5* |
| | [157] | | Mariën et al., case 6 | [170] | | *Semenza et al., case 6* |
| | [158] | | Mariën et al., case 7 | [171] | 2004 | *Bartha et al.* |
| | [159] | | Mariën et al., case 8 | [172] | | *Seddoh et al.* |
| | [160] | | Mariën et al., case 9 | [173] | | *Sheehy and Haines* |
| | [161] | | *Shintani et al.* | [174] | 2006 | *Mansur et al.* |
| | [162] | 2002 | Paghera et al. | [175] | 2007 | *Salis et al.* |
| | [163] | | *Paparounas et al.* | [176] | | *Tabeling et al.* |
| | [164] | 2003 | *Diouf et al.* | | | |

Legend: References printed in cursive cases added till 2007.
References printed in bold reliable subcortical CAD-cases.

Appendix 3: Three epoch time-frame analysis of 9 possible/reliable subcortical CAD-cases with a vascular etiology

| | | | aphasia symptoms | | | | | | severity score and | | |
|---|---|---|---|---|---|---|---|---|---|---|---|
| reference | age/gender | time post-onset | fluency | compreh | repetition | naming | reading | writing | aphasia type | | lesion site |
| [4] Colombo et al., 1984 | 67/M | acute phase | | +++ | +++ | +++ | +++ | +++ | 15 | wernicke | basal ganglia |
| | | lesion phase | | | +++ | | ++ | +++ | 8 | *wernicke* | |
| | | late phase | | | +++ | | ++ | +++ | 8 | *wernicke* | |
| [11] Deleval and Léonard, 1991, case 3 | 58/M | acute phase | +++ | ++ | +++ | +++ | ++ | +++ | 16 | broca | anterior intern capsule, extern |
| | | lesion phase | ? | | + | | | + | ? | | capsule and semi-oval centre |
| | | late phase | ? | ? | | | ? | ? | ? | | |
| [13] Cappa et al., 1993, case 1 | 79/F | acute phase | +++ | +++ | + | +++ | +++ | +++ | 16 | mixed transcortical | periventricular white matter |
| | | lesion phase | +++ | +++ | + | +++ | ++ | ? | 12 | mixed transcortical | and lenticular nucleus |
| | | late phase | ++ | ++ | | ++ | | +++ | 9 | ? | |
| [14] Cappa et al., 1993, case 2 | 56/M | acute phase | | + | | +++ | | | 4 | *anomic* | periventricular region |
| | | lesion phase | | | | | | | | no aphasia | |
| | | late phase | | | | | | | | no aphasia | |
| [15] Gomez-Tortosa et al., 1994 | 38/F | acute phase | | | | | | | 0 | no aphasia | subcortical infarction involving |
| | | lesion phase | | | | | | | 0 | no aphasia | lenticular branches of the middle |
| | | late phase | | | | | | | 0 | no aphasia | cerebral artery |
| [19] Laiacona et al., 1996 | 68/M | acute phase | ? | ? | ? | ? | ? | ? | ? | global | genu and posterior limb of |
| | | lesion phase | +++ | ++ | + | ++ | +++ | +++ | 14 | *transcortical motor* | internal capsule, lateral and posterior |
| | | late phase | ? | ? | ? | ? | ? | ? | ? | | parts of the thalamus and putamen |
| [22] Mariën et al., 2001, case 3 | 75/F | acute phase | ++ | + | + | | + | ++ | 7 | transcortical motor | crus anterior of the internal capsule, |
| | | lesion phase | +++ | + | ++ | | +++ | +++ | 12 | transcortical motor | globus pallidus, putamen, external |
| | | late phase | +++ | + | ++ | | | + | 7 | transcortical motor | capsule, claustrum and periventricular white matter |
| [23] Mariën et al., 2001, case 4 | 79/F | acute phase | +++ | +++ | +++ | +++ | +++ | +++ | 18 | global | parietal white matter and basal ganglia |
| lesion phase | ? | ? | ? | ? | ? | ? | ? | ? | | | |
| | | late phase | ? | ? | ? | ? | ? | ? | ? | ? | |
| *[25] current observation, 2007* | 70/M | acute phase | ? | ? | ? | ? | ? | ? | ? | ? | thalamus |
| | | lesion phase | | +++ | | + | +++ | +++ | 10 | transcortical sensory | |
| | | late phase | | ++ | | + | ++ | | 5 | | |

**Legend:** M male; F female; + + + severely disturbed; ++ moderately disturbed; + mildy disturbed, (blank) not disturbed; ? no information; severity scores (number of + signs) between 1 and 6 indicate mild aphasia, between 7 and 12 moderate aphasia, between 13 and 18 severe aphasia, zero score indicates absence of aphasia; aphasia type printed cursive: interpretation of the authors; reference printed bold and cursive: case added in 2007; compreh = comprehension.

Appendix 4: Neurocognitive symptoms of 9 possible/reliable subcortical CAD-cases with a vasuclar etiology

| reference | age/ gender | dysprosodia | visual neglect | constructional apraxia | ideomotor apraxia | ideational apraxia | oral apraxia | acalculia | additional cognitive disturbances | lesion site |
|---|---|---|---|---|---|---|---|---|---|---|
| [4] Colombo et al., 1984 | 67/M | ? | + | + | + | + | + | ? | anosognosia | basal ganglia |
| [11] Deleval and Léonard,1991, case 3 | 58/M | ? | − | + | − | − | ? | ? | Wechsler PIQ = 90 | anterior intern capsule, extern capsule and semi-oval centre |
| [13] Cappa et al., 1993, case 1 | 79/F | + | + | ? | + | − | + | ? | anosognosia | periventricular white matter and lenticular nucleus |
| [14] Cappa et al., 1993, case 2 | 56/M | + | − | ? | − | ? | − | ? | ? | periventricular region |
| [15] Gomez-Tortosa et al., 1994 | 38/F | ? | − | − | − | − | − | ? | impaired immediate and delayed verbal and visual memory, decreased short term attention and concentration | subcortical infarction involving lenticular branches of the middle cerebral artery |
| [19] Laiacona et al., 1996 | 68/M | + | + | + | − | + | − | + | ? | genu and posterior limb of internal capsule, lateral and posterior parts of the thalamus and putamen |
| [22] Mariën et al., 2001, case 3 | 75/F | + | + | + | − | − | − | ? | none | crus anterior of the internal capsule, globus pallidus, putamen, external capsule, claustrum and periventricular white matter |
| [23] Mariën et al., 2001, case 4 | 79/F | ? | ? | ? | ? | ? | ? | ? | ? | parietal white matter and basal ganglia |
| *[25] current observation, 2007* | 70/M | − | + | + | + | + | − | + | concentration and memory deficits | thalamus |

**Legend:** M male; F female; '+ present'; − absent; ? no information; PIQ performal IQ; reference printed bold and cursive: case added in 2007.
